# Magnetic Fields in Cancer Therapy: Mechanistic Insights, Signaling Pathways, and Evidence from Clinical and In Vitro Studies

**DOI:** 10.3390/pharmaceutics18060742

**Published:** 2026-06-15

**Authors:** Sadettin Berkay Sarli, Asiye Busra Boz Er

**Affiliations:** 1 Department of Physics, Faculty of Science, Karadeniz Technical University, Trabzon 61080, Turkey; sbsarli@ktu.edu.tr; 2 Department of Medical Biology, Faculty of Medicine, Recep Tayyip Erdogan University, Rize 53200, Turkey

**Keywords:** magnetic fields, cancer therapy, magnetic hyperthermia (MHT), pulsed electromagnetic fields (PEMF), magnetic nanoparticles (MNPs), signaling pathways

## Abstract

Magnetic fields (MFs) represent an emerging modality in cancer therapy, encompassing static, low-frequency, pulsed, and nanoparticle-mediated alternating fields. These interventions have demonstrated the capacity to modulate proliferation, apoptosis, ferroptosis, migration, and epithelial-to-mesenchymal transition (EMT) in tumor cells, often through reactive oxygen species (ROS) modulation, ion channel regulation, membrane receptor dynamics, and lysosomal membrane permeabilization. Magnetic nanoparticle hyperthermia (MHT) has reached clinical application, showing promising outcomes in glioblastoma and prostate cancer, while pulsed electromagnetic fields (PEMFs) and magneto-mechanical approaches are under preclinical investigation. The mechanistic diversity of MFs allows synergistic combination with chemotherapy, radiotherapy, and immunotherapy. However, parameter sensitivity, field standardization, and long-term safety remain challenges. Here, we review mechanistic insights, signaling pathways, and experimental and clinical evidence for MF-based cancer therapies, highlighting translational potential and the need for rigorous optimization to realize clinical efficacy.

## 1. Introduction

In contemporary daily life, humans are continuously exposed to magnetic fields (MFs) generated by electricity distribution networks, domestic appliances, communication systems, and transportation infrastructure [1]. While such everyday exposures are generally low in intensity, their potential influence on human health—particularly cancer risk—remains a topic of debate. At the same time, magnetic fields have emerged as engineered tools in oncology, capable of modulating tumor cell behavior when applied under controlled conditions. This duality—ubiquitous environmental exposure versus deliberate therapeutic application—underscores the importance of understanding how MFs interact with biological systems [2].

Magnetic fields used in cancer research and clinical settings can be broadly categorized into several types. Static magnetic fields (SMFs) extend from weak background exposures in the microtesla-to-millitesla range to strong laboratory-generated fields exceeding several tesla [3]. Extremely low-frequency electromagnetic fields (ELF-EMFs), typically oscillating at 50/60 Hz, represent the most common form of environmental exposure [4]. Pulsed electromagnetic fields (PEMFs), already employed clinically to promote bone repair, deliver time-varying stimulation with tunable parameters [5]. Finally, alternating fields applied to magnetic nanoparticles (MNPs) form the basis of magnetic hyperthermia, a therapeutic strategy in which localized heating selectively damages tumor tissue, often enhancing the effects of chemotherapy or radiotherapy [6].

At the cellular and molecular level, MFs have been shown to regulate a wide array of cancer-related processes. Depending on field characteristics and cell type, they can modulate proliferation, arrest cell-cycle progression, and trigger apoptosis or ferroptosis, frequently through pathways linked to oxidative stress and mitochondrial dysfunction [7]. They may also influence autophagy, alter reactive oxygen species (ROS) homeostasis, and perturb calcium signaling, membrane potential, and cytoskeletal organization, thereby affecting cell migration and epithelial-to-mesenchymal transition (EMT)-like behavior. However, these effects are highly parameter-dependent—varying with intensity, frequency, waveform, and duration—and are therefore often inconsistent across studies [8].

From a public health standpoint, the omnipresence of ELF-EMFs raises valid concerns regarding long-term cancer risk, though epidemiological studies have thus far produced inconclusive results [9]. Most laboratory data suggest that effects at environmental levels are mild and reversible, though subtle contributions to tumorigenesis over long timescales cannot be excluded. By contrast, translational research has embraced controlled magnetic fields as therapeutic agents. Among these, MNP-mediated hyperthermia represents the most advanced approach, with clinical trials demonstrating promising tumor regression through localized heating while sparing surrounding tissues [10]. Moreover, emerging evidence indicates that magnetic fields may potentiate conventional modalities, including chemotherapy, radiotherapy, and immunotherapy, thereby positioning MFs as promising adjuvant strategies [11].

Magnetic fields can interact with tumors either indirectly via engineered transducers (magnetic nanoparticles that convert fields into heat or force) or directly when low-frequency fields perturb electrophysiology and stress pathways [12]. MHT has progressed furthest clinically, with intratumoral iron-oxide nanoparticle instillation followed by AMF exposure showing feasibility and meaningful survival signals in recurrent glioblastoma when combined with stereotactic radiotherapy, and interstitial thermotherapy explored in localized prostate cancer [13,14,15].

A separate translational thread is magnetic drug targeting (MDT), in which chemotherapeutics or carriers are steered to tumor vasculature by external magnets [16]. The first-in-human phase I feasibility study used intra-arterial mitoxantrone with magnetic targeting and reported selective localization with preliminary antitumor activity [17,18].

On the cell-biology side, robust studies in breast and cervical cancer lines document PEMF-mediated modulation of viability and drug response and magneto-mechanical lysosomal disruption capable of rapidly inducing apoptosis—mechanisms complementary to, and in some cases orthogonal to, thermal cytotoxicity [14,19,20].

**Static Magnetic Fields (SMF)**: Emerging evidence shows that moderate to strong SMFs (ranging from ~150 mT to several tesla) can modulate cancer cell behavior through non-thermal, magnetomechanical or orientation-dependent mechanisms. For instance, exposure to ~150 mT SMF in 4T1 breast cancer cells accelerated proliferation but inhibited migration and telomerase activity, implicating biomechanical orientation effects and transcriptional regulation [21]. Similarly, high-intensity SMFs directly altered the orientation of EGFR kinase domains, leading to reduced proliferation in EGFR-overexpressing cells—supporting a direct physical influence on protein conformation [22].

**Pulsed Electromagnetic Fields (PEMF)**: Low-frequency PEMFs (<100 Hz, mT-level) typically exert magnetomechanical effects on ion channels and signaling pathways, without generating significant heat. In breast cancer cell lines (e.g., MCF-7, MDA-MB-231), 8 Hz PEMFs at ~0.011 T reduced proliferation and induced senescence selectively in cancer cells while sparing normal fibroblasts, illustrating a non-thermal, selective cytotoxic effect [23]. In vivo, PEMF exposure enhanced CD8^+^ T-cell cytotoxicity and inhibited cervical cancer progression via the SMAD3/p38/NF-κB axis, further highlighting immune–mechanical crosstalk [24].

**Alternating Magnetic Fields (AMF) for Magnetic Hyperthermia (MHT)**: AMFs in the ~100 kHz range engage magnetothermal mechanisms through nanoparticle heating, directly inducing tumor cell death via apoptosis, necrosis, or enhanced immunogenicity. Magnetic nanoparticle hyperthermia is well characterized in oncology, and has shown strong potential for spatially localized thermal ablation and synergy with other therapies [11,25].

**Figure 1:**
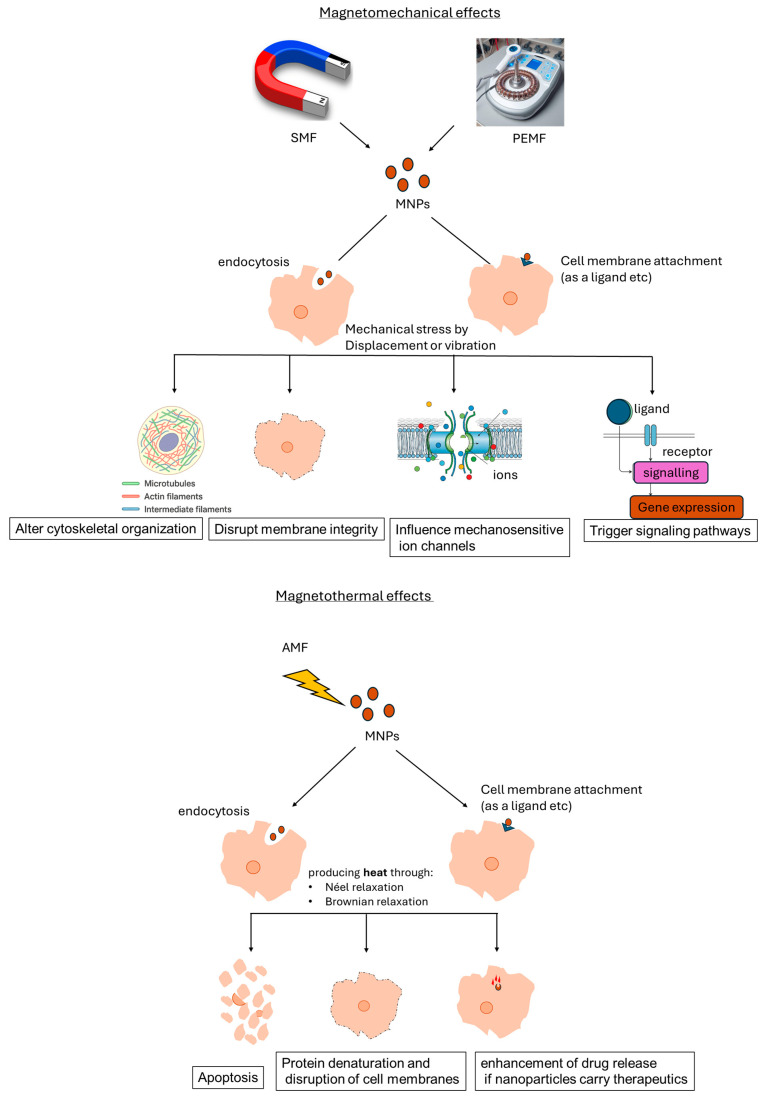
Schema of Magnetomachanical effects and Magnetothermal effects [26,27,28,29,30,31,32,33,34,35].

Despite progress, the biological and clinical implications of magnetic fields remain scattered, and a systematic classification is missing. To address this gap, this review categorizes magnetic field applications in cancer therapy according to three principal types: static magnetic fields (SMF), pulsed electromagnetic fields (PEMF), and alternating magnetic fields (AMF). Within this framework, we distinguish two major mechanisms of action: (i) magnetomechanical (MM) effects, where mechanical forces generated by magnetic nanoparticles or field gradients perturb cellular processes, and (ii) magnetothermal (MT) effects, where alternating fields induce localized heating for hyperthermia-based therapies (Figure 1). This classification provides a systematic foundation to link biological outcomes with field parameters and application scenarios. Specifically, SMFs influence cell proliferation and cytoskeletal organization, PEMFs modulate apoptosis and immune activation via non-thermal mechanisms, and AMFs primarily engage magnetothermal effects for hyperthermia. This framework underpins the structure of the present review.

This review aims to systematically evaluate the biological effects of magnetic fields and magnetic nanoparticles on cancer cells, with a focus on parameter-dependent outcomes and translational potential. By linking biophysical principles to biological responses, we highlight opportunities and challenges for integrating magnetic modalities into future therapeutic strategies.

## 2. Biological Effects of Magnetic Fields on Cancer Cells

**Figure 2:**
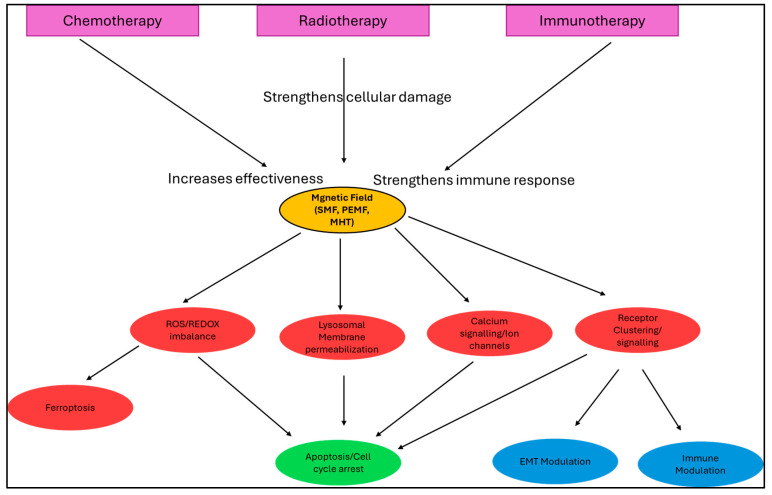
An overview of the mechanisms by which magnetic fields influence cancer cells and their potential integration with existing cancer therapies [20,41,42,43,44,45,46,47,48].

Static Magnetic Fields (SMFs) show nuanced effects on proliferation and migration. For example, exposure to 1 T SMF reduced viable cell numbers in several solid human cancer cell lines—particularly at high cell densities—likely through modulation of the EGFR–Akt–mTOR survival pathway [36]. Another study found that moderate SMFs (~150 mT) applied to 4T1 breast cancer cells inhibited migration and telomerase activity, while increasing proliferation—a potential trade-off involving telomere regulation [21]. Additionally, SMFs directly affected the orientation of EGFR kinase domains in an intensity-dependent manner (up to 9 T), thereby inhibiting proliferation in EGFR-overexpressing cancer cells—supporting a physically oriented, non-thermal mechanism (Figure 2) [22].

Pulsed Electromagnetic Fields (PEMFs) primarily modulate calcium signaling and apoptosis. In mesenchymal stem cells, PEMF stimulated proliferation and differentiation through enhanced intracellular Ca^2+^ transients and activation of MAPK signaling pathways [37]. In MCF-7 breast cancer cells, PEMFs (2.5 mT at 70 Hz for repeated exposures) heightened etoposide-induced cell death via increased ROS generation, DNA damage response, and caspase-dependent apoptosis [38].

Alternating Magnetic Fields (AMFs), particularly in the context of magnetic hyperthermia, drive oxidative stress–mediated tumor cytotoxicity. Human hepatocarcinoma cells exposed to AMF (around 186 kHz) in the presence of Fe_3_O_4_ nanoparticles showed significant increases in oxidative DNA damage markers (8-oxo-dG) in a dose- and time-dependent manner [39]. Similarly, oral cancer cells treated with cisplatin and ferucarbotran (SPIONs) under AMF (~308 kHz) exhibited pronounced hyperthermia (~42.5 °C) and enhanced apoptosis compared to cisplatin alone [40].

### 2.1. Proliferation and Cell-Cycle Regulation

Many primary studies report that magnetic-field effects on proliferation are parameter- and cell-type dependent. Moderate-to-strong static magnetic fields (SMFs) have been shown to alter proliferative signalling: exposure to 1 T SMF modulated the EGFR–Akt–mTOR axis and reduced proliferation in several cancer cell lines under specific conditions, an effect that depended on cell density and receptor expression [22]. Conversely, moderate SMFs (~150 mT) accelerated proliferation yet suppressed migration and telomerase activity in murine 4T1 breast cancer cells, illustrating how the same nominal field can produce divergent outcomes depending on biological context [21]. Together these studies underscore that claims about “SMF increases or decreases proliferation” must be qualified by field strength, exposure regimen, and cell type [22]. In MCF-7 cells, exposure to 0.1–1 mT ELF-MF increased proliferation via ERK activation [49,50]. In contrast, stronger fields promoted apoptosis, underscoring safety concerns.

### 2.2. Apoptosis, Autophagy and Ferroptosis

Apoptosis and autophagy are frequent readouts in EMF studies and often reflect non-thermal (PEMF/SMF) or nanoparticle-mediated thermal/mechanical mechanisms. Pulsed electromagnetic fields (PEMFs) can potentiate chemotherapeutic apoptosis: for example, PEMF co-exposure enhanced etoposide-induced ROS accumulation, DNA damage and caspase-dependent apoptosis in MCF-7 cells [38]. Separately, magnetically actuated nanoparticles under alternating magnetic fields (AMF/MHT) elicit thermal and microthermal stresses that activate apoptosis or autophagy depending on dose, particle loading and thermal history [51,52]. Importantly, emerging work shows that magnetic strategies can also trigger ferroptosis-like death via MF-boosted Fenton chemistry or nanoparticle iron release, providing an orthogonal cell-death route particularly relevant when iron-containing carriers are used [53].

### 2.3. Oxidative Stress (ROS) and Redox Homeostasis

Modulation of reactive oxygen species (ROS) is a recurrent mechanistic thread linking diverse MF exposures to downstream outcomes. PEMF and SMF regimens have been reported to increase intracellular ROS in cancer cells, amplifying DNA damage and apoptotic signalling in supportive contexts, while other exposure paradigms elicit adaptive antioxidant responses instead—again highlighting parameter sensitivity [38]. In nanoparticle-assisted AMF treatments, localized heating and/or nanoparticle degradation further elevate labile iron and ROS production, which can potentiate lipid peroxidation and ferroptotic pathways when combined with disrupted antioxidant defence [51,53].

Recent studies show that MF-induced calcium influx via voltage-gated and mechanosensitive channels exacerbates mitochondrial depolarization, thereby accelerating ROS generation [54]. ROS can directly trigger lysosomal membrane permeabilization (LMP) through lipid peroxidation, linking calcium signalling, oxidative stress, and lysosomal instability into a single interconnected cascade [55].

### 2.4. Calcium Signalling, Ion Channels and Membrane Perturbation

Several primary studies directly connect low-frequency EMFs and PEMFs to changes in intracellular Ca^2+^ dynamics and ion-channel function. ELF/PEMF exposures have been shown to increase cytosolic Ca^2+^ transients and activate Ca^2+^-sensitive signalling (FAK/Rho GTPases, MAPKs) that regulate migration, differentiation and cell fate in multiple cell types [56,57]. Mechanistically, these calcium effects may arise from modulation of TRP channels, voltage-gated channels, or altered membrane receptor clustering; in cancer cells, such perturbations can shift the balance between proliferation, apoptosis and motility depending on exposure parameters [56].

Magneto-mechanical forces on membranes may activate Piezo1/TRPV channels, causing Ca^2+^ influx [58,59]. Moreover, disruption of integrin clustering by mechanical perturbation alters downstream signalling, providing a mechanistic link between LMP, ion fluxes, and receptor signalling [60,61].

### 2.5. Migration, Invasion, and EMT Modulation

Magnetic fields can alter cytoskeletal organization and adhesive behaviour, producing anti-metastatic phenotypes in some models. For example, rotating low-frequency magnetic fields (0.1–0.4 T at ~4.2 Hz) directly destabilized F-actin and reduced migration, invasion and metastatic colonization of breast cancer cells in vitro and in vivo, consistent with a magnetomechanical mode of action [62]. Likewise, moderate SMF exposure (≈150 mT) suppressed migration and telomerase function in 4T1 cells, despite concurrent proliferative effects, illustrating that migration/EMT readouts frequently diverge from proliferation endpoints under the same MF regimen [21]. These studies suggest magnetic interventions may be particularly useful to target motility and metastatic processes, but emphasize the need to couple EMT/migration assays with detailed parameter reporting [62].

## 3. Mechanistic Models of Magnetic Field Action in Cancer Research

Building upon the basic interactions between magnetic fields and cellular components described above, the following section discusses their impact on cell-cycle regulation and survival pathways. Several mechanistic models have been proposed to explain how magnetic fields (MFs) exert their effects on cancer cells. One prominent hypothesis involves reactive oxygen species (ROS)-mediated damage. MFs, particularly when combined with iron or magnetic nanoparticles (MNPs), can enhance ROS production, leading to oxidative damage of DNA and proteins. This in turn can trigger apoptosis, ferroptosis, or cellular senescence [63,64]. Another mechanism is based on magneto-mechanical effects, where oscillating magnetic fields acting on MNPs generate physical forces that stress cellular membranes or intracellular organelles, disrupt the cytoskeleton, and ultimately alter cell viability or motility [65]. A third proposed mechanism involves ion-channel modulation and calcium signaling, as magnetic fields may influence the gating of voltage-gated or T-type calcium channels. Such alterations can propagate downstream effects on signal transduction and transcriptional regulation [50]. Finally, the most well-understood mechanism is the thermal effect. Alternating magnetic fields cause MNPs to generate localized heating, producing hyperthermia (≥41 °C), which leads to protein denaturation, membrane disruption, and cell death [66]. Importantly, magnetic hyperthermia also exerts immunomodulatory effects and represents the clearest, dose-dependent, and physically characterized mechanism, one that has already advanced into clinical application and trials.

High-frequency AMFs (typically in the 100–300 kHz range) are optimized for magnetothermal effects in magnetic hyperthermia (Table 1). This frequency range balances safety with heating efficiency, as reflected in specific absorption rates (SAR) and the Rosensweig power equation [67]. For instance, some studies report substantial heat generation in Fe_3_O_4_ nanoparticle suspensions when exposed to 100 kHz fields, especially at field amplitudes of a few millitesla [67,68].

Strong field gradients (greater than 10 T/m) substantially enhance MM forces, such as nanoparticle displacement or membrane stress. In diabetic mouse models, highly non-uniform SMFs with gradients > 10 T/m induced increased ROS and apoptosis, while uniform SMFs did not elicit such effects [69]. Theoretical frameworks similarly suggest that gradients may alter transmembrane potentials or ion-channel function via mechanical stress [65].

The choice of waveform, frequency, and duty cycle can bias biological outcomes. For example, PEMFs at 8 Hz and 11 mT selectively induced apoptosis in breast cancer cells while sparing fibroblasts [23]. Low-frequency PEMFs (1 mT) reduced ROS and inflammatory cytokine levels, whereas higher frequencies (6–7 mT) increased ROS, DNA damage, and apoptosis, illustrating parameter-dependent effects on cell fate [70].

### 3.1. ROS-Mediated Damage

One of the most frequently proposed models of magnetic field (MF) action in cancer biology involves the modulation of reactive oxygen species (ROS) [71]. Exposure to pulsed electromagnetic fields (PEMFs), extremely low-frequency electromagnetic fields (ELF-EMFs), or magnetic nanoparticle-mediated alternating magnetic fields has been shown to increase intracellular ROS production [72]. This elevation in ROS can overwhelm the antioxidant defense systems of cancer cells, which are often already under oxidative stress due to rapid proliferation and metabolic reprogramming [73]. The resulting oxidative damage to DNA, lipids, and proteins can trigger apoptosis, ferroptosis, or permanent cell-cycle arrest. Importantly, because malignant cells are more vulnerable to redox imbalance than healthy cells, this mechanism suggests a potential therapeutic window for selective cancer targeting. At the same time, excess ROS can also promote mutations and genomic instability, raising the possibility that magnetic field exposure might have dual outcomes depending on the context and duration of exposure. Whether ROS drives apoptosis or tumorigenesis depends on dose, duration, and repair capacity. Transient bursts promote cell death, whereas chronic sub-lethal ROS foster mutagenesis and adaptive resistance [74,75]. Thus, understanding how MF exposure balances between cytotoxicity and tumor-promoting mutagenesis is crucial for its translation into cancer therapy [76,77,78].

### 3.2. Ferroptosis and Redox Integration

Magnetic fields (MFs) influence the redox state of cancer cells by altering radical pair lifetimes and modifying mitochondrial electron flow [73]. When iron is present—either as endogenous labile Fe^2+^ or in the form of superparamagnetic iron oxide nanoparticles (SPIONs)—Fenton chemistry intensifies, amplifying lipid peroxidation and pushing cells toward ferroptosis [79]. The ferroptosis threshold is tightly regulated by factors such as GPX4, FTH1, SLC7A11, ACSL4, and intracellular iron pools. In this context, MF exposure can create parameter regimes where reactive oxygen species (ROS) accumulate without sufficient NRF2-driven antioxidant compensation, thereby sensitizing tumors to chemotherapy, radiotherapy, or magnetic hyperthermia (MHT) [80,81,82]. Conversely, robust NRF2 or HO-1 induction may blunt cytotoxicity and promote resistance. Practical biomarkers for monitoring these processes include oxidative DNA lesions (8-oxo-dG), lipid peroxidation products (4-HNE, MDA), mitochondrial membrane potential (Δψm), glutathione ratios (GSH/GSSG), BODIPY-C11 oxidation, and measures of transferrin receptor or labile iron pool levels [71,83,84]. These readouts can help stratify tumor types most vulnerable to MF-driven ferroptosis.

### 3.3. Lysosomal Membrane Permeabilization (LMP) via Magneto-Mechanics

A unique aspect of SPION-based therapies is their preferential accumulation within endo-lysosomal compartments [85]. Under oscillating or alternating magnetic fields, these nanoparticles can undergo mechanical torque or translation, physically stressing lysosomal membranes [20]. This local mechanical disruption triggers lysosomal membrane permeabilization (LMP), releasing cathepsins into the cytosol and activating apoptotic cascades independent of bulk temperature rise [85]. This mechanism explains observations of rapid apoptosis even when average tissue heating is minimal, distinguishing mechanical MF effects from pure thermal damage [85]. LMP also synergizes with DNA-damaging therapies, providing a complementary cell-death pathway [86]. Experimental tracking can rely on galectin-3 puncta formation, loss of LysoTracker fluorescence, detection of cytosolic cathepsin B/L, and pharmacological rescue using lysosomal inhibitors such as bafilomycin A1 [87,88]. Together, these approaches underscore LMP as a distinct and therapeutically exploitable mechanism of MF-induced cytotoxicity.

### 3.4. Membrane Receptor Modulation and Signaling Dynamics

Another line of evidence suggests that magnetic fields may directly influence the organization and activity of membrane proteins, particularly receptors involved in survival and proliferation pathways [89]. Cancer cells rely heavily on receptor tyrosine kinases (RTKs), integrins, and G-protein-coupled receptors (GPCRs) to sense their microenvironment and sustain malignant signaling [90]. Magnetic fields, through physical effects on lipid bilayer fluidity or via the orientation of charged residues, have been reported to modulate receptor clustering and downstream signaling cascades [91]. For example, PEMFs have been associated with altered activity of EGFR and integrin β1, leading to disruptions in focal adhesion signaling and PI3K/AKT pathway activation [92,93,94]. Such effects can impair cell adhesion, migration, and metastatic potential, while sensitizing tumor cells to conventional therapies. However, the same mechanisms may also induce adaptive responses, including compensatory activation of alternative receptors or pathways, which could contribute to treatment resistance. This dual potential highlights the importance of dissecting receptor-specific effects when integrating MF-based interventions into oncology.

### 3.5. Calcium Signaling and Ion Channel Regulation

Magnetic fields are also hypothesized to affect the function of ion channels, particularly those regulating calcium flux across cellular membranes [95,96]. Calcium is a master regulator of diverse cellular processes, including proliferation, apoptosis, and migration [97]. Evidence suggests that ELF-EMFs and PEMFs can alter intracellular calcium dynamics by modulating voltage-gated calcium channels, transient receptor potential (TRP) channels, or store-operated calcium entry (SOCE) mechanisms [56,98,99]. In cancer cells, where calcium homeostasis is often dysregulated to favor survival and invasion, MF-induced perturbations can tip the balance toward cell death or differentiation [100]. For instance, enhanced calcium influx may activate pro-apoptotic calpain or caspase pathways, while disrupting cytoskeletal organization critical for invasion [101]. Conversely, under certain conditions, increased calcium signaling might promote proliferation and EMT, underscoring the context-dependent nature of MF effects [102]. Thus, calcium-mediated pathways represent both an opportunity and a caution in leveraging MF exposure, demanding precise control over exposure parameters to maximize therapeutic benefit.

### 3.6. Immune Modulation and Tumor Microenvironment Interactions

Beyond direct effects on tumor cells, magnetic fields may also influence the tumor microenvironment (TME) and immune surveillance [2]. Tumor-associated macrophages, dendritic cells, and T lymphocytes have all been shown to respond to EMFs through altered cytokine secretion and activation states [103,104,105]. For instance, exposure to PEMFs can increase pro-inflammatory cytokines such as TNF-α and IL-6, enhancing anti-tumor immune responses, while also modulating the phagocytic activity of macrophages [106,107,108]. Similarly, magnetic nanoparticle-based hyperthermia has been reported to induce immunogenic cell death, releasing damage-associated molecular patterns (DAMPs) that activate dendritic cells and prime cytotoxic T cells [109]. This suggests that MFs may synergize with immune checkpoint inhibitors or adoptive T-cell therapies by reshaping the TME into a more immunostimulatory state. Nonetheless, inappropriate or chronic magnetic field exposure may drive macrophage polarization toward an M2-like, tumor-promoting state and potentiate immunosuppressive signaling within the tumor microenvironment, thereby counteracting anti-tumor immunity [110]. Therefore, immune modulation represents one of the most promising—yet complex—dimensions of MF-based cancer therapy, offering opportunities for combination strategies with immunotherapy.

Exposure of macrophages or macrophage co-cultures to pulsed electromagnetic fields or low-frequency magnetic fields has been shown to modulate polarization toward anti- or pro-inflammatory phenotypes depending on the MF parameters. For example, Vinhas et al. (2020) showed that a PEMF exposure in co-culture of IL-1β-primed tendon cells with THP-1-derived macrophages increased expression of the anti-inflammatory marker IL-10, upregulated M2-associated genes (like MRC-1) and downregulated pro-inflammatory markers such as NOS-2 and TNF-α [111].

On the other hand, in “Innate Immune Regulation Under Magnetic Fields with Possible Mechanisms and Therapeutic Applications” (2021), various studies are summarized in which low-frequency alternating magnetic fields (LF-MFs) increase pro-inflammatory cytokines (e.g., IL-1β, IL-6, TNF-α) in RAW264.7 or bone marrow-derived macrophages, depending on field strength and frequency. Moderate or high gradient magnetic fields (HGMFs), by contrast, tend to bias macrophage polarization toward the anti-inflammatory M2 phenotype, often associated with increased expression of IL-10 and other “repair/regulatory” markers [112].

These findings suggest that subtle differences in MF amplitude, frequency, exposure duration, and/or gradient can determine whether macrophages adopt tumor-promoting (M2-like) or tumor-suppressive (M1-like) roles. Mechanistically, changes in signaling pathways like STAT6 upregulation and suppression of STAT1 have been implicated in the M2 polarization under certain MF conditions [112].

## 4. Experimental and In Vitro Evidence

Experimental evidence from in vitro models further supports the potential of MFs in oncology. For example, pulsed electromagnetic fields (PEMFs) have been shown to potentiate chemotherapy. In MCF-7 breast cancer cells, PEMF exposure enhanced etoposide-induced cell death through a mechanism involving ROS accumulation, DNA damage, and activation of caspase-dependent apoptotic pathways [23,38]. Another consistent line of evidence comes from magnetic nanoparticle–mediated hyperthermia, where iron oxide nanoparticles exposed to alternating magnetic fields reliably induce cell death through localized heating. Numerous studies have demonstrated that this approach not only kills cancer cells directly but also sensitizes them to radiotherapy and chemotherapy, while imaging-guided systems further improve spatial precision and safety [25]. In contrast, the effects of static magnetic fields (SMFs) appear more variable and highly intensity-dependent (Table 2). Some studies report that specific SMF intensities can inhibit proliferation and suppress epithelial-to-mesenchymal transition (EMT), while others observe negligible or even stimulatory effects. High-field exposures in the tesla range tend to produce distinct responses compared to lower-intensity exposures, highlighting the complexity of MF–cell interactions [113,114].

## 5. Translational Applications

Among translational applications, magnetic hyperthermia (MHT) remains the most mature and clinically advanced [11]. Preclinical and clinical studies have shown that MNPs activated by alternating magnetic fields can induce local tumor regression, with applications currently being explored in breast, prostate, and brain cancers, among others. Advances in imaging-guided MHT and the development of multifunctional nanoparticles for combined therapeutic and diagnostic (“theranostic”) purposes are further expanding the scope of this approach [25]. In addition, PEMF and low-frequency electromagnetic devices are under active investigation. Several experimental systems are being designed to selectively inhibit tumor growth or to enhance tumor sensitivity to conventional drugs [119]. Early preclinical studies have produced encouraging results; however, successful clinical translation will require careful standardization of exposure parameters, improved understanding of mechanisms, and comprehensive safety evaluation [120].

On the biophysical side, several well-established logical tests can be applied to distinguish between magnetomechanical (MM) and magnetothermal (MT) effects. The following measures provide a systematic approach for resolving this distinction.

First, according to the principle of macro-scale heat generation, if bulk suspension temperature measurements—obtained Via calorimetry or thermal imaging—show a significant rise, MT effects are likely responsible for the observed outcomes [121]. Conversely, if cell death occurs without a corresponding bulk temperature increase, this suggests MM contributions (e.g., localized mechanisms or surface heating).

Following the evaluation of macro thermal changes, the particle immobilization (gelation) test can be employed. When the same particles are immobilized in a viscous gel, disappearance of the AC-field-induced effect indicates a predominant MM (Brownian) contribution. In contrast, persistence of thermal elevation under these conditions reveals a dominant MT (Néel) mechanism [122]. Because this strategy has been consistently applied in numerous studies, it is now considered a standardized and essential diagnostic test [123,124,125,126].

Thirdly, the test for AC susceptibility ($\chi^{\prime \prime }(f)$) measurement can be performed in conjunction with frequency scanning. In AC magnetic susceptibility ($\chi^{\prime }$, $\chi^{\prime \prime }$) measurements, the loss component $\chi^{\prime \prime }$ typically exhibits a peak frequency ($f_{p}$) that is related to the relaxation time. In the event that the peak frequency $\chi^{\prime \prime }$ is in close proximity to the applied frequency $f_{p}$ and the $P\propto H^{2}f$ relationship is observed, this result is indicative of an MT (LRT) effect [127,128,129].

Furthermore, the fourth test is examined using low-frequency (Hz–kHz) application and imaging (live confocal). This test, conducted at low frequencies (10–100 Hz) and corresponding magnetic field strengths, suggests a high probability of MM effect in response to observed intracellular aggregate/lysosome movement/rupture. Also, Néel relaxation is ineffective because energy loss at these frequencies is negligible. However, when intracellular lysosomal movement, membrane deformation or rupture is observed, energy transfer occurs not thermally but through direct magnetomechanical (MM) interactions [123,130,131,132].

Immobilization and temperature-sensitive biomarkers (e.g., ROS generation vs. mechanical fragments) have been proposed in the literature as a fifth diagnostic test for distinguishing between MT and MM effects. Predominance of thermal stress markers—such as heat shock proteins (HSPs) or a generalized heat response—indicates an MT-driven mechanism. In contrast, evidence of oxidative stress, lysosomal leakage, or mechanical membrane rupture supports the involvement of MM processes [133,134,135].

The field dependence test (sixth test) is generally scalable with $\propto H^{2}$ for both MM and MT effects. However, aggregation, magnetic interactions, and non-linear behaviour exhibit divergent *H* thresholds in the MM case. Consequently, simultaneous frequency/field scanning and immobilisation testing are mandatory [19,136,137,138,139].

### 5.1. Magnetic Hyperthermia Therapy (MHT)

Magnetic hyperthermia therapy (MHT) exploits the ability of superparamagnetic iron oxide nanoparticles (SPIONs) to generate localized heat when exposed to an alternating magnetic field (AMF). This effect arises from Néel and Brownian relaxation losses, which lead to the dissipation of thermal energy in the tumor microenvironment, thereby inducing apoptosis, necrosis, or sensitization to chemotherapy and radiotherapy [140,141,142]. Clinical translation of MHT has been most successful in glioblastoma multiforme (GBM), where the FDA-approved NanoTherm^®^ therapy demonstrated improved survival when used in combination with radiotherapy [141,143]. Prostate cancer trials have similarly shown promising safety and efficacy. Advances in theranostic nanoparticle design now allow SPIONs to serve dual functions: acting as contrast agents for magnetic resonance imaging (MRI) while simultaneously functioning as localized heat sources. Moreover, integration with image-guided delivery systems enhances spatial precision and minimizes off-target effects, making MHT a powerful adjunct to conventional cancer therapies [85]. These clinical and preclinical successes highlight the importance of understanding the physical principles underlying MHT and optimizing experimental parameters [140].

The physical concept underlying MHT studies are based on Rosensweig power calculations [129]. In small-angle (ξ≪1) and linear response assumptions, the average power per unit volume (suspension) is given by,

(1) $$P_{V}=\frac{1}{2}\mu_{0}\omega {H_{0}}^{2}\chi^{\prime \prime }\left(\omega \right).$$

Here, ω=2πf, $\mu_{0}$ is the permeability of the vacuum, the expression $\chi^{\prime \prime }\left(\omega \right)$,

(2) $$\chi^{\prime \prime }\left(\omega \right)=\chi_{0}\frac{\omega \tau }{1+\omega^{2}\tau^{2}}$$

τ is the effective relaxation time (below), and $\chi_{0}$ is the low-frequency sensitivity. For independent SPIONs, $\chi_{0}$ is $\chi_{0}=\mu_{0}nm^{2}/3k_{B}T$, $m=M_{s}V_{p}$ and $n=\varphi /V_{p}$ (φ: volume fraction, $V_{p}$: single particle volume) is given; this Equation (2) reduce to

(3) $$\chi_{0}=\frac{\mu_{0}{M_{s}}^{2}\varphi V_{p}}{3k_{B}T}$$

From Equation (3) it is observable that relaxation mechanisms operate in two modes: while it is defined as Brownian relaxation caused by metal nanoparticles rotating around their own axis as first mode. Second mode is known Néel relaxation, which occurs when the external magnetic field effect is lost, and magnetic moment/internal spin motion [144]. Néel relaxation is defined as,

(4) $$\tau_{N}=\frac{1}{2}\tau_{0}\sqrt{\frac{\pi k_{B}T}{KV}}e^{KV/k_{B}T}.$$

Here, $\tau_{0}$ is called characteristic relaxation time [145,146] and takes a value in the $\tau_{0}\approx 10^{-9}\sim 10^{-13}\;s$. Brownian relaxation time is calculated from,

(5) $$\tau_{B}=\frac{3\eta V_{H}}{k_{B}T}.$$

Overall, final relaxation time τ can be calculated,

(6) $$\frac{1}{\tau }=\frac{1}{\tau_{B}}+\frac{1}{\tau_{N}}$$

using these two relaxation times. These calculations allow researchers to predict and optimize the effects of experimental parameters, such as particle size, field strength, and frequency, on MHT efficacy. Ultimately, this approach ensures the effective and safe application of MHT in both clinical and preclinical settings [147,148]. The relaxation times derived from the calculations are utilised in the particle immobilisation test, which is then presented in comparison with Néel and Brownian.

### 5.2. Magnetic Drug Targeting (MDT)

Magnetic drug targeting (MDT) represents another application of magnetic nanoparticles, where drug-loaded SPIONs are guided to tumor sites using external magnetic fields. Early feasibility studies demonstrated that mitoxantrone, an anthracenedione chemotherapeutic, could be magnetically localized in experimental tumor models, thereby increasing intratumoral accumulation while reducing systemic toxicity [149]. This approach has since been expanded to a variety of drug–nanoparticle conjugates, with evidence showing enhanced therapeutic indices and reduced off-target cytotoxicity [150]. Despite these encouraging results, the primary limitation of MDT remains the depth and precision of magnetic field penetration in human tissues, restricting its practical application largely to superficial or organ-confined tumors. For typical permanent magnet arrays, clinically achievable targeting depths are limited to ~2–3 cm [151]. Advanced superconducting or Halbach array designs may extend this to ~5 cm, but remain technically challenging. Ongoing research on implantable magnetic field-generating devices and advanced magnet arrays shows potential to overcome these limitations and improve clinical translatability [152].

From a physical perspective, MDT is conceptualised based on classical magnetic force $\vec{F}$,

(7) $$\vec{F}=\vec{\nabla }(\vec{m}\cdot \vec{B})\approx \frac{V∆\chi }{2\mu_{0}}\vec{\nabla }(B^{2})$$

calculations for magnetic targeting (the magnetic force exerted on a nanoparticle) as linear regime (non-saturation) $\vec{F}$. Here, $∆\chi =(\chi_{p}-\chi_{m})$ is called susceptibility difference between in-phase $\chi_{p}$ and out off-phase $\chi_{m}$. In the presence of magnetic saturation, $m\to m_{s}=M_{s}V$ and Equation (7) simplifies to,

(8) $$F\approx m_{s}\left|\vec{\nabla }\vec{B}\right|$$

Equation (8) stokes drag is also calculated using the force, $F_{D}=6\pi \eta av$, when there is flow towards the target. The condition for the capture process, which is required to ensure target adhesion, is $F≳F_{D}$ [153,154]. These equations provide a quantitative framework for designing and optimizing MDT experiments, enabling precise targeting of therapeutic nanoparticles while minimizing off-target distribution.

Overall, these physical principles provide a quantitative foundation for optimizing MDT strategies. By carefully selecting nanoparticle properties, magnetic field strengths, and exposure geometries, researchers can maximize intratumoral accumulation while minimizing systemic dispersion. Such optimization is crucial for translating MDT from preclinical models to human patients, particularly for tumors located at challenging depths or within organs. Continued advances in nanoparticle engineering, field-guiding devices, and real-time imaging modalities promise to enhance the precision, safety, and therapeutic efficacy of MDT, potentially establishing it as a versatile complement to conventional cancer treatments.

### 5.3. Magneto-Mechanical Approaches

Beyond thermal and targeting strategies, magneto-mechanical therapy leverages the torque and oscillatory forces exerted by magnetic nanoparticles under an alternating or rotating magnetic field. These mechanical forces can induce cell membrane rupture, disrupt cytoskeletal architecture, and trigger apoptotic pathways, representing a distinct cytotoxic mechanism independent of hyperthermia [141]. Preclinical studies have demonstrated that oscillating magnetic fields acting on anisotropic nanoparticles, such as nanorods or nanodiscs, can selectively kill cancer cells while sparing healthy counterparts [140,141]. Although still in the experimental stage, these approaches are particularly appealing because they offer an orthogonal strategy to conventional thermal methods and may be especially useful for overcoming resistance to hyperthermia or chemotherapeutic treatments.

The fundamental equation of torque is utilised in these magneto-mechanical calculations, which is defined as $\vec{\tau }=\vec{m}\times \vec{B}$ classically based on the magnetic moment $\vec{m}=M_{s}V\hat{e}$. In order to solve for the rotational friction coefficient (assumed sphere, $\zeta_{rot}\;=\;{8\pi \eta r}^{3}$) in a viscous medium, as outlined in $\vec{\tau }$, it is first necessary to assume that the magnetic field *B* required to synchronously drive the rotational $f_{rot}$ at the maximum stable angular velocity $\omega_{rot}=\tau /\zeta_{rot}$ is calculated and then used as $B_{sync}\approx \zeta_{rot}(2\pi f_{rot})/m$ [42,155,156,157]. These calculations provide a quantitative basis for designing magneto-mechanical therapies, allowing researchers to predict the required field strengths and frequencies to achieve effective cytotoxic mechanical forces.

### 5.4. Pulsed Electromagnetic Field (PEMF) Devices and Low-Frequency Applications

**Figure 3:**
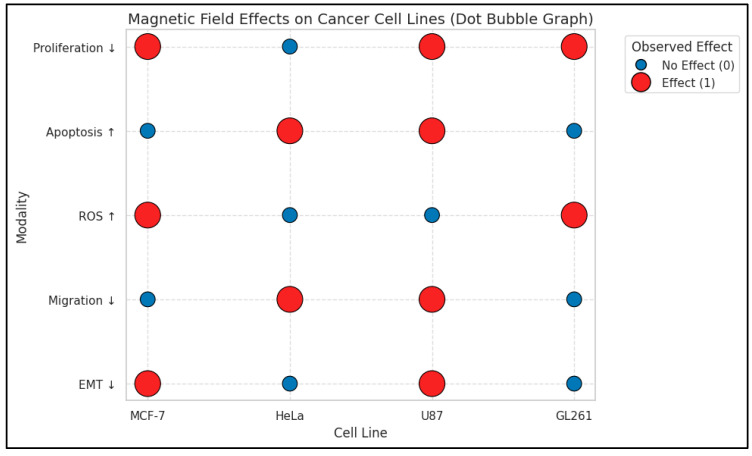
Magnetic Field Effects on Cancer Cell Lines. The dot bubble graph summarizes the observed effects of magnetic field exposure on various biological modalities across different cancer cell lines. *X*-axis represents the cancer cell lines (MCF-7, HeLa, U87, GL261). *Y*-axis represents the biological modalities [38,161,162,163,164,165]. ↑: upregulation, ↓: downregulation.

In addition to nanoparticle-mediated approaches, pulsed electromagnetic fields (PEMFs) and low-frequency electromagnetic fields have been investigated for their direct biological effects on tumor cells. Preclinical studies have reported that exposure to PEMFs can reduce cancer cell proliferation, promote apoptosis, and sensitize tumor cells to chemotherapeutic agents such as doxorubicin and cisplatin [158,159]. These effects are thought to be mediated through modulation of reactive oxygen species (ROS), intracellular calcium signaling, and p53 activation (Table 3), (Figure 3) (see Supplementary Materials). Despite encouraging laboratory data, translation into standardized clinical protocols remains limited by the lack of consensus on optimal exposure parameters, including frequency, intensity, and treatment duration [160]. The development of precise dose–response maps and mechanistic biomarkers will be crucial for advancing PEMF therapies into mainstream oncology.

**Table 3:** In vitro studies of magnetic field effects on cancer cells.

Modality	Cell Line(s)	Field Parameters	Key Findings
PEMF	MCF-7, MDA-MB-231	50 Hz, 1 mT	Reduced proliferation, increased apoptosis Via ROS and p53 activation [38]
SMF	HeLa, MCF-7	1 T static	Decreased growth rate, enhanced cisplatin cytotoxicity [113]
MHT	U87, GL261	100 kHz, SPIONs	Induced mitochondrial apoptosis, reduced tumor growth in mice [166,167]
Magnetomechanical	Jurkat, MDA-MB-231	1 kHz, NP torque	Mechanical membrane disruption, caspase activation [165,168]

## 6. Clinical Translation and Trials

### 6.1. Recurrent Glioblastoma

The largest prospective experience comes from intratumoral iron-oxide nanoparticle thermotherapy combined with fractionated stereotactic radiotherapy. In a two-center, single-arm study of 66 patients (of whom 59 had recurrent glioblastoma), median OS from first recurrence was 13.4 months (95% CI 10.6–16.2), with moderate side effects and no serious complications reported. A prior feasibility study established procedural safety, temperature monitoring, and local control signals [13].

Ongoing work aims to optimize applicators and field delivery and to integrate MHT with modern chemoradiation and immunotherapy. Selected updates and reviews summarize device advances and combination strategies [140,169].

### 6.2. Prostate Cancer

Interstitial thermotherapy for prostate cancer has been explored in phase I settings, demonstrating feasibility, patient-specific 3-D thermal planning, and the need to manage discomfort at higher field strengths; early studies also integrated brachytherapy [14].

### 6.3. Human Feasibility of Magnetic Drug Targeting

The first-in-human MDT trial administered intra-arterial mitoxantrone with magnetic targeting.

In advanced solid tumors, demonstrating selective localization and feasibility; this remains the most often cited human MDT feasibility evidence [170] (Table 4).

Magnetic nanoparticle hyperthermia is a cancer treatment where tiny iron particles are placed into a tumor and then heated by an alternating magnetic field. The heat can damage cancer cells or make them more sensitive to treatments like radiotherapy.

It has been tested mainly in glioblastoma (a type of brain cancer) and prostate cancer. Early studies showed it is safe and can improve survival compared with older data. Larger registries and clinical trials are still running to confirm how well and how safely it works (Table 4).

## 7. Limitations

While MHT has reproducible human data in glioblastoma and prostate feasibility settings, larger randomized trials are limited. Standardization of field parameters, nanoparticle dosing, and reporting will be key for cross-study comparisons. The requirement for high-field gradient magnets, RF coils, and cooling systems significantly raises costs. Device miniaturization and energy efficiency remain barriers for clinical adoption [174]. For PEMF, parameter sensitivity (frequency, amplitude, duty cycle) and tumor-specific biology likely explain divergent results; robust dose–response mapping and biomarker-guided designs (e.g., DDR competence, lysosomal stability) are priorities. Magneto-mechanical strategies are compelling but remain preclinical; translation will hinge on targeting, field penetration, and safety [175].

Long-term or high-intensity exposures may have off-target effects; while magnetic hyperthermia targets tumors, systemic MNP biodistribution, long-term iron handling, and heating of non-target tissue require careful preclinical toxicology [25]. Regulatory assessment must consider both electromagnetic exposure and nanoparticle safety.

From the perspective of classical electromagnetic physics, in the context of alternating magnetic fields (AMFs), the generation of eddy currents within conductive tissues arises directly from Faraday’s law. The tissue’s electrical conductivity (σ), together with the frequency (f) and amplitude (H) of the magnetic field, plays a critical role in determining the strength of these currents. This interaction induces thermal effects, resulting in localized temperature elevation within the targeted tissue regions.

At elevated frequencies or large field amplitudes, the thermal consequences for the patient can become significant, potentially causing tissue damage, pain, burns, or even systemic effects [176]. Heat diffusion in this context is constrained by factors such as blood perfusion and the intrinsic thermal properties of tissues—specifically, electrical conductivity, specific heat capacity, and thermal conductivity.

From a safety standpoint, “non-critical” heating thresholds are applied, commonly defined as maintaining local tissue temperatures at 42–43 °C for a controlled duration and ensuring that increases in core body temperature remain within safe limits [122,177,178].

The Brezovich (Atkinson–Brezovich) limit, derived from experimental studies by Atkinson and Brezovich, specifies that H·f < 4.85 × 10^8^ A·m^−1^·s^−1^ [179,180]. In certain cases, this limit has been extended slightly to H·f < 5 × 10^8^ A·m^−1^·s^−1^ for regions of small volume or diameter. This guideline serves two primary purposes: (1) minimizing patient discomfort, burns, and systemic heat load during prolonged exposures; and (2) preventing both local and systemic tissue injury [176].

However, it is important to note that this limit is idealized, derived primarily from experiments on healthy individuals and designed for large-diameter body regions. In more localized applications—such as intracranial treatments or small tumor sites—higher H·f values may be tolerable if both the affected tissue volume and body region diameter are sufficiently small [181,182].

## 8. Future Directions

Magnetic field-based cancer therapies offer considerable promise, yet significant challenges remain before their widespread clinical adoption. Standardization of exposure parameters—including frequency, intensity, waveform, and duration—is critical to achieve reproducible and predictable biological effects across tumor types. Integrating magnetic field modalities with chemotherapy, radiotherapy, or immunotherapy may enhance therapeutic efficacy, but requires careful mechanistic understanding to prevent adaptive resistance. Advances in nanoparticle design and targeted delivery are needed to maximize tumor specificity, enable simultaneous imaging and therapy, and overcome limitations of tissue penetration. Elucidating mechanisms such as ROS modulation, lysosomal membrane permeabilization, receptor clustering, and calcium signaling will help identify predictive biomarkers (Potential predictive biomarkers include ROS scavenging enzymes (SOD2, GPX4) [183,184], lysosomal cathepsins, mechanosensitive ion channels (Piezo1, TRPV4 [185,186]), and macrophage polarization markers (CD163/CD206 vs. CD80/CD86) [187,188]) and responsive tumor subtypes. Additionally, optimizing magnetic field-induced immune modulation to enhance antitumor immunity while avoiding immunosuppressive polarization represents a promising avenue for combination with immunotherapies. Comprehensive safety evaluation—including nanoparticle biodistribution, off-target heating, and long-term effects—is essential, and regulatory frameworks must consider both electromagnetic exposure and nanoparticle safety. For example, Yarjanli et al. reported iron accumulation and oxidative stress in brain tissue 6 months post-SPION exposure [189]. Surface coatings (dextran, PEG) reduce but do not eliminate persistence. Beyond conventional superparamagnetic iron oxide nanoparticles (SPIONs), alternative iron-oxide-based nanoparticle classes present unique opportunities and challenges for in vivo biomedical applications. Natural magnetosomes—magnetite (Fe_3_O_4_) crystals produced by magnetotactic bacteria—exhibit stable single-domain magnetic behavior and very high specific absorption rates, enabling more efficient magnetic hyperthermia than many synthetic SPIONs of comparable size [190]. Their native lipid bilayer membrane confers excellent biodegradability and low cytotoxicity, but reproducibility and large-scale production remain difficult [191]. To address these hurdles, biomimetic iron oxide nanoparticles have been synthesized to mimic magnetosome size and composition (~40–60 nm), offering tunable surface chemistry and scalable fabrication strategies. For example, the use of magnetosome-associated proteins such as MamC or Mms6 enables precise control of particle morphology and magnetic properties [192], while polymersome-based approaches yield magnetosome-like vesicles with comparable intrinsic loss power for hyperthermia [193]. SPIONs, though the most widely studied and commercially available, often show lower heating efficiency and may aggregate in vivo, necessitating careful coating or clustering strategies to improve performance and biocompatibility [194]. A critical comparison of these nanoparticle classes underscores the need for standardized evaluation of magnetic behavior, immune compatibility, and long-term biodistribution to optimize their clinical translation. Finally, large, randomized clinical trials with standardized protocols and biomarker-guided patient stratification are needed to validate efficacy, reproducibility, and translational potential, ultimately enabling magnetic field-based interventions to become robust, clinically actionable cancer therapies.

## Figures and Tables

**Table 1:** Summary of key magnetic field types, their primary physical parameters, and associated biological or mechanistic outcomes. AMF (alternating magnetic field, MHT: magnetic hyperthermia therapy).

Magnetic Field Type	Key Parameters	Biological/Mechanistic Outcome	
AMF (MHT)	Frequency: 100–300 kHz, H limited by safety; $H\cdot f<5\times 10^{9}\;A/m\cdot s^{-1}$	Efficient localized heating, tumor cell death via hyperthermia (MT)	[67,68]
SMF (Static)	Gradient: >10 T/m vs. uniform fields	Enhanced ROS and apoptosis with high gradients; uniform fields show no strong effect	[69]
PEMF	Frequency: low (1 mT) vs. high (6–7 mT); Duty cycle & waveform (e.g., 8 Hz, square wave)	Low frequency dampens ROS; high frequency increases apoptosis; selective cancer cell cytotoxicity	[23,70]

**Table 2:** Summary of magnetic field-based modalities in cancer therapy. The table lists the typical frequency or field strength, delivery methods, primary mechanisms of action, and current clinical status of each approach. Abbreviations: MHT, magnetic nanoparticle hyperthermia; AMF, alternating magnetic field; PEMF, pulsed electromagnetic fields; SMF, static magnetic fields.

Magnetic Field Type	Parameters	Cell/Model	Observed Effect	Mechanism (Magnetomechanical/Magnetothermal)	Reference
**SMF**	150 mT, continuous	4T1 breast cancer cells	Accelerated proliferation; inhibited migration; decreased telomerase activity	Magnetomechanical	[21]
**SMF**	1 T, continuous; high cell density	Human cancer cell lines (various)	Reduced proliferation; EGFR–Akt–mTOR pathway altered	Magnetomechanical	[36]
**SMF**	0.26–0.33 T, gradient 2.09 T/m, 72 h	MCF-7 & HeLa cells	Reduced Young’s modulus and adhesion; altered F-actin	Magnetomechanical	[115]
**PEMF**	50 Hz, 10 mT, 3–6 h	Rat mesenchymal stem cells	Enhanced proliferation; G1-phase increase	Magnetomechanical	[116]
**PEMF**	30 Hz, 1 mT, 2 h/day, 3 d	C3H10T1/2 mesenchymal cells	Ca^2+^-dependent osteoblastogenesis Via Wnt/Ca^2+^ signaling	Magnetomechanical	[117]
**PEMF (rotating)**	0.1–0.4 T at 4.2 Hz	Breast cancer model	Disrupted F-actin; reduced migration/invasion; suppressed metastasis in vivo	Magnetomechanical	[62]
**AMF (MHT)**	~300 kHz, Fe_3_O_4_ nanoparticles	Hydrogel model	Heat generation 43–47 °C depending on Fe_3_O_4_ content	Magnetothermal	[51]
**AMF (MHT)**	—	MCF-7 cells + Mn oxide NPs + AMF	Apoptosis with minimal temperature rise (<0.5 °C)	Magnetothermal	[118]
**AMF**	Not specified	Fe_3_O_4_ NP hyperthermia	Effective heating in ferrogel for hyperthermia	Magnetothermal	[51]

**Table 4:** Key clinical studies using magnetic fields in cancer therapy.

Modality	Field Type/Params	Cancer	Design/Phase	Primary Outcome	Key Result (Short)	Registry/Pub
Magnetic nanoparticle hyperthermia (NanoTherm)	Alternating MF ~100 kHz; 2–15 kA/m	Recurrent glioblastoma	Single-arm; combined with RT (phase I/II)	Overall survival after recurrence	Feasible; reported OS improvement vs. historical controls	[13] PMC3097345
Magnetic nanoparticle hyperthermia (NanoTherm) VALIDATE	Alternating MF (device-specific)	Recurrent glioblastoma	Prospective registry; multicenter	Median survival vs. SoC (registry comparison)	Ongoing/registry; aims to validate efficacy & safety	DRKS00023339 [171]
Magnetic nanoparticle hyperthermia (NanoTherm)—Adjuvant	Alternating MF (device-specific)	Glioblastoma (adjuvant setting)	ClinicalTrials.gov listing	Efficacy & tolerance	Active/registered trial	NCT06271421 [172]
Magnetic nanoparticle hyperthermia (interstitial)	Alternating MF; ~4–18 kA/m	Locally recurrent prostate cancer	Feasibility/pilot (with MRI thermometry)	Ability to reach hyperthermia; safety	Feasible; hyperthermic–thermoablative temps achieved	[173] (Eur Urol)

## Supplementary Materials

The following supporting information can be downloaded at: https://www.mdpi.com/article/10.3390/pharmaceutics18060742/s1, File S1: Dot bubble graph generation, Data source and preparation.
