# Supplementary material for: Magnetic Fields in Cancer Therapy: Mechanistic Insights, Signaling Pathways, and Evidence from Clinical and In Vitro Studies
Source: Pharmaceutics. 2026 Jun 15;18(6):742. doi: 10.3390/pharmaceutics18060742 (PMC13306740; doi:10.3390/pharmaceutics18060742)
Supplement: Supplementary file 1 [file pharmaceutics-18-00742-s001.zip › pharmaceutics-3866266-supplementary.pdf]

9. Supplementary

9.1. Dot Bubble Graph Generation

To visually represent the observed effects of magnetic field exposure on various cancer cell lines, a dot bubble graph was generated using Python (version 3.x), leveraging the pandas (version 1.x), matplotlib (version 3.x), and seaborn (version 0.x) libraries.

9.1.1. Data Source and Preparation

The input data summarized the presence (1) or absence (0) of specific biological effects (proliferation decrease, apoptosis increase, reactive oxygen species increase, migration decrease, and epithelial-to-mesenchymal transition decrease) across four distinct cancer cell lines (MCF-7, HeLa, U87, and GL261). This data was initially structured in a wide format, with cell lines as columns and biological modalities as rows. For visualization, the data was transformed into a long format (also known as "melted" format) using pandas.melt(). This conversion created a dataset where each row contained a unique combination of 'Modality', 'Cell Line', and 'Effect' (value of 0 or 1), making it suitable for seaborn's plotting functions.

9.1.2. Visualization Parameters

Custom visual mappings were defined to clearly differentiate between the two states of effect:

- **Color Mapping:** An effect value of '0' (no effect observed) was mapped to a dark blue color, while an effect value of '1' (effect observed) was mapped to a dark red color.
- **Size Mapping:** To further emphasize the presence of an observed effect, a smaller dot size was assigned to '0' values, and a proportionally larger dot size was assigned to '1' values.

9.1.3. Graph Generation

The scatterplot was generated using seaborn.scatterplot(), with 'Cell Line' mapped to the x-axis and 'Modality' mapped to the y-axis. The hue and size arguments were both linked to the 'Effect' variable, applying the defined custom color and size mappings. Black edgecolor was added to each dot to enhance visual distinctiveness.

9.1.4. Figure Formatting

The plot was enhanced with a descriptive title ("Magnetic Field Effects on Cancer Cell Lines (Dot Bubble Graph)"), clear x and y-axis labels, and a grid for improved readability. A custom legend was manually created and added to the plot to explicitly define the meaning of the dot colors and sizes, ensuring comprehensive interpretation of the visual representation. The layout was adjusted using plt.tight_layout() to prevent any overlap of labels or elements.

9.2. AI Usage

Chatgpt 5.0 and Grammarly used for grammar corrections, and Googlecolab used for the generation of graphs.
